# The role of primary and secondary delays in the effective resonance frequency of acoustically interacting microbubbles

**DOI:** 10.1016/j.ultsonch.2022.106033

**Published:** 2022-05-13

**Authors:** Hossein Haghi, Michael C. Kolios

**Affiliations:** a Ryerson University, 350 Victoria Street, Toronto, Ontario, Canada; b Institute for Biomedical Engineering, Science and Technology (iBEST), a partnership between St. Michael’s Hospital and Ryerson University, 209 Victoria St, Toronto, Ontario, Canada

**Keywords:** Bubble dynamics, Cavitation, Acoustic cavitation, Bubble-bubble interaction, Microbubbles

## Abstract

Acoustically excited microbubbles (MBs) are known to be nonlinear oscillators with complex dynamics. This has enabled their use in a wide range of applications from medicine to industry and underwater acoustics. To better utilize their potential in applications and possibly invent new ones a comprehensive understanding of their dynamics is required. In this work, we explore the effect of bubble-bubble interactions on the resonance frequency of MB suspensions. MBs oscillate in response to an external acoustic wave and since bubbles in a cluster are at different locations compared to the excitation source, they are excited at different times. In this work we refer to these delays as primary delays. Interactions between the scattered pressure fields from adjacent bubbles have also been shown to alter the dynamics of MBs that exist within clusters. These secondary waves generated by MBs reach MBs in their proximity at different times that depend on their spatial location in the cluster. Here we refer to these delays as secondary delays. Inclusion of the secondary delays modifies the class of the differential equations governing the oscillations of interacting MBs in a cluster from ordinary differential equations to neutral delay differential equations. Previous work has not considered the all the delays associated with the bubble distances when modeling the interactions between bubbles. In this work we investigate the effect of both the primary and secondary delays on the effective resonance frequency of MB clusters. It is shown that primary delays cause spreading the resonance frequency of identical MBs within a range where the closest MB to the acoustic source exhibits the lowest resonance frequency and the furthest MB resonates at the highest frequency. This range has been shown to be up to 0.12 MHz for the examples investigated in this work. The effect of secondary delays is shown to be very significant. In the absence of secondary delays, the ordinary differential equation model predicts a decrease of up to 26% in the resonance frequency of 4 identical interacting MBs as the inter-bubble distances are decreased. However, we show that inclusion of the secondary delays result in the increase of the resonance frequency of MBs if they are situated close to each other. This increase is shown to be significant and for the case of 4 identical interacting MBs we show an increase of 58% in the resonance frequency.

## Highlights

• Primary and secondary delays of within microbubble (MB) clusters is introduced.
• Primary delays spread the resonance frequency of identical MBs within a range.
• The closest MB to the ultrasound source resonates at the lowest frequency.
• The furthest MB from the ultrasound source resonates at the highest frequency.
• Secondary delays cause the resonance frequency of MBs to increase with concentration.

## 1 Introduction

MBs excited with ultrasonic waves are known to be nonlinear oscillators [1], [2], [3], [4], [5] Their rich and complex oscillatory dynamics have been utilized in a wide range of applications ranging from industry to medicine. Numerous studies have been conducted to better understand the physical behavior of MBs. Moreover, the nonlinear response of MBs in response to ultrasound waves has been the basis for their use as ultrasound contrast agents for blood flow measurement [6], [7]. Under certain conditions [8], MBs have been shown to be capable of violent oscillations producing powerful shockwaves which have been utilized in breaking histotripsy [9] and lithotripsy [10]. Sonoluminescence, a phenomenon in which MBs can produce very localized high temperatures accompanied by a flash of light has shown potential to be used in sonochemical reactors [11]. Controlled stable oscillations of MBs generate micro-streaming in the surrounding liquid which can be used to pump or stir liquid at miniature scales [12]. The same MB oscillations have been utilized in medicine for site specific drug and gene delivery [13] through sonoporation [14], [15], reversibly opening the blood brain barrier [16], [17], [18] and to increase drug uptake in proximity of active oscillating MBs [19].

To maximize the efficacy of MBs in their applications and possibly conceive new ones, a comprehensive understanding of their complex dynamics is necessary. The radial oscillations of ultrasonically excited MBs have been shown to depend on variety of parameters, some native to physical properties of the MBs in use (physical properties of encapsulating shell or lack thereof, size, gas content, etc.), some dependent on the medium in which bubbles exist (medium density, surface tension, viscosity of liquid, etc.) and some relevant to the properties of the exciting ultrasound wave (frequency, pressure amplitude, etc.). One of the basic acoustic properties of MBs is their resonance frequency ($f_{r}$)[7]. The resonance frequency of MBs is an important parameter in determining the optimized ultrasound exposure parameters to maximize the efficacy of the MB oscillations. When sonicated at their resonance frequencies, the energy transfer from ultrasound wave to MBs is maximized and consequently the scattering cross-section and therefore scattered ultrasound signal [20], [21] are also maximized. Moreover, the resonance frequency of MBs have been a parameter of interest to help optimize exposure parameters for non-destructive oscillations: MBs are exhibit stable non-destructive oscillations when sonicated at a frequency equal to twice their resonance frequency [22].

The resonance frequency of MBs depends on several factors. These parameters include the viscosity of host media [23], physical properties of the encapsulating shell (or lack thereof) [24], [25], [26], the initial radius of the MB [27], pressure amplitude of the applied acoustic wave [28], geometry of the MB’s surrounding such as the boundaries of a blood vessel wall [29], [30] or near a rigid wall [31] or attached to an elastic wall [32]. In this work we show that inter-bubble interactions play an important role in determining the resonance frequency of MBs. The majority of applications utilizing MBs employ them in clusters. MBs oscillating in response to an incoming acoustic wave (the primary wave) generate secondary acoustic waves within the media. Therefore, within a MB cluster, each MB oscillates in response to the primary and the secondary acoustic waves. Therefore, a MB cluster is a collection of coupled nonlinear oscillators where the coupling is done through the secondary acoustic waves in the media.

Since an ultrasound wave has a finite speed, different bubbles within a bubble cluster are exposed to the incident waves at different times which is determined by their spatial location (distance from transducer). We refer to this delay between excitation of bubbles as the primary delay (since the primary ultrasound wave is delayed between different bubbles). In addition, oscillating bubbles generate secondary pressure waves within the liquid which also travel at a finite speed (speed of sound) within the media. The secondary waves also reach neighboring bubbles at different times with a delay dependent on their distances from the bubble source of the secondary wave. These delays are referred to as secondary delays in this manuscript.

For a two-bubble system and the case of bubble chains, it has been shown that the inclusion of time delay results in higher damping coefficients for lower frequencies where the lowest frequency mode exhibits the highest damping [33]. In a recent study [34], a theoretical model accounting for time delays was experimentally validated through a spatially asymmetrical acoustic emissions from an ultrasonically excited bubble cloud. It has been shown that coupling in MB clusters can modify the resonance frequencies of MBs [35], [36], [37]. In [38] it has been shown that with increasing concentration of MBs (therefore reducing inter-bubble distances) and improving the coupling between MBs, their resonance frequency decreases. In most studies, due to the small inter-bubble distances, the effect of time delays is assumed to be insignificant and neglected [38], [39]. For instance in [40] authors examine the subharmonic emissions of interacting MBs without inclusion of delays in their investigation and conclude that the subharmonic resonance frequency decreases with strengthening inter-bubble interactions. In [41], the authors examined the dynamics as well as the translational motion of interacting bubbles (without the inclusion of delays) which experience viscoelastic drag in a viscoelastic media.They found the elasticity of the media to be a key parameter for the translational motion of MBs and that the viscoelasticity of the media prevents MBs from moving in the space.

In [42], the authors developed a model for translational motion of two interacting bubbles which excludes the effect of delays. The model proposed in [42] results in discrepancies with experimental data in [43]. In [44] authors attribute the discrepancies between their experimental and numerical results on the movement of ultrasonically excited MBs in a tube to the sliding friction between the MBs and the tube walls. However, in [43] the authors eliminated this possibility by making sure that MBs are far from walls in their proximity and concluded the discrepancies observed in their experiments and in [44] is not due to sliding friction on the walls. The authors suggest the need for a more precise model for the translational dynamics of interacting pulsating MBs.

The aim of this study is to investigate effect of primary and secondary delays on the dynamics of bubbles as a cluster. More specifically, this study focuses on examining the fundamental relevance and the effect of primary and secondary delays on the effective linear resonance frequency of interacting MBs. To achieve this goal, we eliminated other known sources of nonlinearity in the bubble dynamics (such as the shell [24], thermal energy dissipation [45], mass transfer [46] etc.), we conduct our numerical study on free (no shell) air bubbles with no thermal dissipation and mass transfer. This enables us to identify the effects we observe as a result of primary and secondary delays which is within the goal of this study. The previously mentioned complexities will be examined in future studies. To ensure that the level of coupling between the MBs is identical, we employed MBs of equal initial radius in our study. Moreover, we chose spatial formations where bubbles are equidistant from each other. This limits our numerical study to a maximum of 4 bubbles (located at the edges of a tetrahedron). Addition of a 5th bubble causes the inter-bubble distances to be unequal. This adds another level of complexity to our numerical study which we avoid so as to analyze the effect of primary and secondary delays.

## 2 Methods

### 2.1 The bubble model

**Fig. 1:**
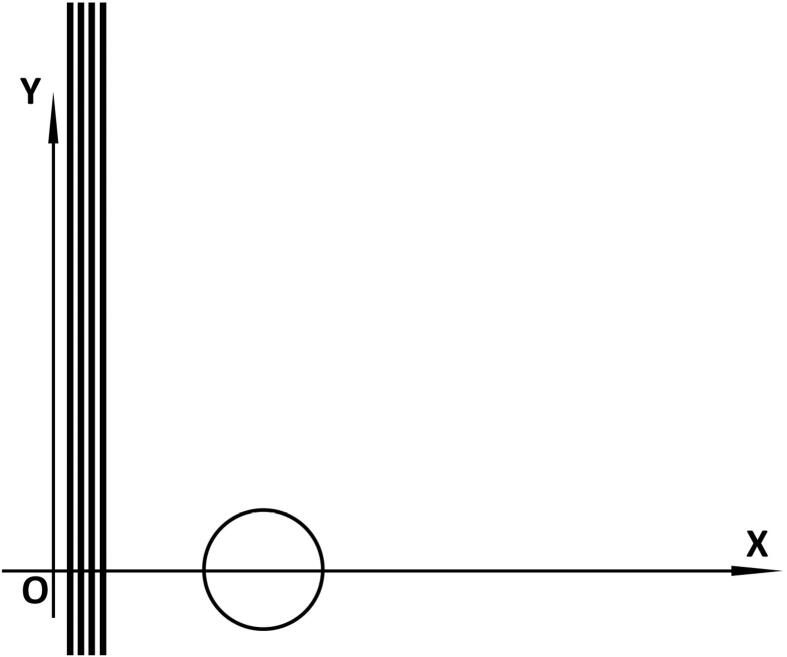
Schematic of a single bubble being sonicated with an ultrasound wave travelling along x-axis.

Fig. 1 shows a single isolated bubble in an infinite domain of liquid sonicated by a plane monochromatic ultrasound wave travelling along the x-axis. The dynamics of this bubble can be described through Keller-Miksis [47] equation as:

(1) $$\left(1-\frac{\overset{˙}{R}}{C_{l}}\right)R\overset{¨}{R}+\frac{3}{2}{\overset{˙}{R}}^{2}\left(1-\frac{\overset{˙}{R}}{C_{l}}\right)=\left(1+\frac{\overset{˙}{R}}{C_{l}}+\frac{R}{C_{l}}\frac{d}{\mathrm{dt}}\right)\frac{P_{B}(R,\overset{˙}{R},t)}{\rho_{l}}$$

where the term $P_{B}$ stands for pressure at the bubble wall and can be written as:

(2) $$\begin{array}{c} P_{B}=\left[1+\frac{\overset{˙}{R}}{c}+\frac{R}{c}\frac{d}{\mathrm{dt}}\right]\left[\left(P_{\infty }+\frac{2\sigma }{R_{0}}\right){\left(\frac{R_{0}}{R}\right)}^{3k}-\frac{2\sigma }{R}-\frac{4\mu_{L}\overset{˙}{R}}{R}-P_{\infty }+P_{a}\sin\left(2\pi \mathrm{ft}-\mathrm{kx}\right)\right] \end{array}$$

Table 1 shows what each parameter in Eqs. (1), (2) represent.

**Table 1:** Parameter definitions.

R	Bubble radius
$\overset{˙}{R}$	Bubble wall velocity
$\overset{¨}{R}$	Bubble wall acceleration
$R_{0}$	Bubble initial radius
$C_{l}$	Speed of sound in liquid (water)
$\rho_{l}$	Liquid (water) density
$\mu_{L}$	Liquid (water) viscosity
σ	Liquid (water) – gas (air) interface surface tension
γ	Specific heat ratio of gas (air) inside
$P_{\infty }$	Ambient pressure
$P_{a}$	Pressure amplitude of the incoming acoustic wave
f	Frequency of the incoming acoustic wave
t	Time
k	Wave number
x	Center of bubble on x axis

In Eq. (2) the term *kx* within $P_{a}\sin\left(2\pi \mathrm{ft}-\mathrm{kx}\right)$ accounts for the primary delays. Keller and Kolodner [48] shown that successive compression and expansion of a spherical cavity in an acoustically compressible media ($\rho_{l}$ constant) will emit a pressure field, the amplitude of which at a distance d away from the center of the sphere can be calculated from Eq. (3)

(3) $$P_{\mathrm{rd}}=\rho_{l}\left(\frac{2R\overset{˙}{R}+\overset{¨}{R}{\overset{˙}{R}}^{2}}{d}\right)$$

Eq. (3) can be used to couple the dynamics of N bubbles in bubbly media as:

(4) $$\begin{array}{c} \left(1-\frac{\overset{˙}{R_{i}}}{C_{l}}\right)R_{i}\overset{¨}{R_{i}}+\frac{3}{2}{\overset{˙}{R_{i}}}^{2}\left(1-\frac{\overset{˙}{R_{i}}}{C_{l}}\right)=\left(1+\frac{\overset{˙}{R_{i}}}{C_{l}}+\frac{R_{i}}{C_{l}}\frac{d}{\mathrm{dt}}\right)\frac{P_{B}(R_{i},\overset{˙}{R_{i}},t)}{\rho_{l}}\\ -\overset{N}{\underset{j=1,j\neq i}{\sum }}\left(\frac{2R_{j}\overset{˙}{R_{j}}+\overset{¨}{R_{j}}{\overset{˙}{R_{j}}}^{2}}{d_{\mathrm{ij}}}\right) \end{array}$$

In its current form, Eq. (4) assumes an infinite sound speed for the re-radiated pressure waves from the bubbles in the media. To address this, we can include a delay in the summation term in Eq. (4) as

(5) $$\begin{array}{c} \left(1-\frac{\overset{˙}{R_{i}}}{C_{l}}\right)R_{i}\overset{¨}{R_{i}}+\frac{3}{2}{\overset{˙}{R_{i}}}^{2}\left(1-\frac{\overset{˙}{R_{i}}}{C_{l}}\right)=\left(1+\frac{\overset{˙}{R_{i}}}{C_{l}}+\frac{R_{i}}{C_{l}}\frac{d}{\mathrm{dt}}\right)\frac{P_{B}(R_{i},\overset{˙}{R_{i}},t)}{\rho_{l}}\\ -\overset{N}{\underset{j=1,j\neq i}{\sum }}\left(\frac{2R_{j}(t-\tau_{\mathrm{ij}})\overset{˙}{R_{j}}(t-\tau_{\mathrm{ij}})+\overset{¨}{R_{j}}(t-\tau_{\mathrm{ij}})\overset{˙}{R_{j}}{\left(t-\tau_{\mathrm{ij}}\right)}^{2}}{d_{\mathrm{ij}}}\right) \end{array}$$

The term $\tau_{\mathrm{ij}}$ (secondary delay) is equal to $d_{\mathrm{ij}}/C_{l}$ where $d_{\mathrm{ij}}$ is the distance between centers of ith and jth bubble.

**Fig. 2:**
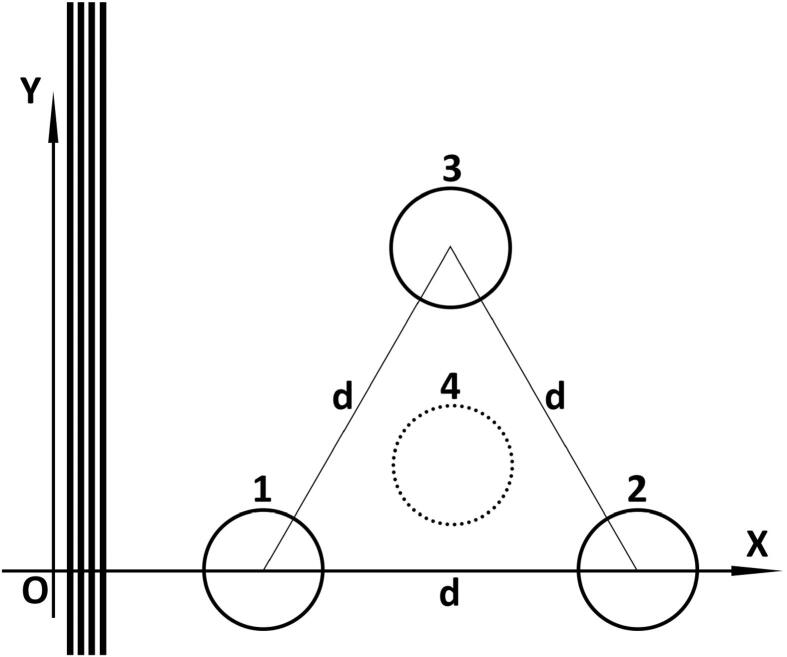
Schematic of multiple MBs situated in a 3D space (dotted bubble is d away from the rest, outside of the page) being sonicated with a plane ultrasound wave travelling along x-axis.

Fig. 2 shows the different geometries that we will be solving the bubble dynamics equations for. The 2nd bubble (B-2) is added at a distance of d from the first bubble. Similarly, third bubble (B-3) is located at a distance of d away from both B-1 and B-2. Following this method with can place a fourth bubble (B-4) at a distance of d away from all the rest. It should be noted that B-4 is not on the X-Y plane and is located outside of it. For simplicity, we assume that B-1 is located at x=0.

### 2.2 Numerical Method

Three different classes of equations are solved for this study as follows:

1-Single bubble
  For this the case of a single bubble, Eq. (1) is solved (a second order ordinary differential equation (ODE). The initial conditions are:
  (6) $$\left\{\begin{array}{c} R(t=0)=R_{0}\\ \overset{˙}{R}(t=0)=0 \end{array}\right)$$
  Eq. (6) describes the bubble at rest at t=0 with a wall velocity of zero ($\overset{˙}{R}=0$) and its initial radius $R_{0}$. Since the bubble (B1) is placed at x = 0, the primary delay in this case is zero. After attempting to solve Eq. (1) with different available numerical packages (e.g. MATLAB), due to its efficiency and speed we chose Tsitouras 5/4 (Tsit5) Runge-Kutta method in DifferentialEquation.jl package of Julia programming language [49]. Julia is described as a fast dynamic language for technical computing [50] where it combines the accessibility of higher level languages such as MATLAB with speed of lower level languages such as C++. For instance, the Tsit5 algorithm in Julia was able to solve the ODE presented in Eq. (1) approximately 50× faster than the ODE45 solver in MATLAB where both solvers use the same mathematical principles.
2-Multiple bubbles with and without primary delays
  For this case, Eq. (4) is solved where the excitation term includes the primary delay for the model with primary delays. Eq. (4) is a system of coupled ODEs with known initial conditions as:
  (7) $$\left\{\begin{array}{c} R_{i}(t=0)=R_{i0}\\ {\overset{˙}{R}}_{i}(t=0)=0 \end{array}\right)$$
  Eq. (7) states that at t = 0, all of the bubbles are at rest and their initial radius is known. Similar to the case of the single bubble, the Tsit5 algorithm is used to solve Eq. (4).
3-Multiple bubbles with primary and secondary delays
  By adding secondary delays to Eq. (4) (with primary delays), Eq. (5) results, which is a system of second order delayed differential equations. A slightly different numerical method is required to solve Eq. (5). The terms inside the summation term are delayed at t=0 and their values at a negative time ($t-\tau_{\mathrm{ij}}<0$ at t=0) are required to solve Eq. (5). This is done through defining a history function for the delayed terms as follows:
  (8) $$\left\{\begin{array}{c} R_{i}(t<0)=R_{i0}\\ {\overset{˙}{R}}_{i}(t<0)=0\\ \overset{¨}{R_{i}}(t<0)=0 \end{array}\right)$$
  Eq. (8) describes bubbles of known initial radius to be at rest (wall velocity and acceleration are zero) before the sonication starts at t = 0. Using the described history function and a DDE solver, the Tsit5 algorithm in the Julia programming language can be used to solve Eq. (5).

Using the methods mentioned above the radial oscillations of the bubbles as a function of time can be obtained. The linear resonance frequency of the bubbles can be calculated by following the steps below:

1-Sonicate bubbles at a low pressure of 1 kPa for 80 cycles across for a range of frequencies
2-Find the maximum amplitude of oscillations in the last 20 cycles (after the transient phase)
3-Map the maximum amplitude of oscillatory radius to the frequency.

More details of this method can be found in Section 2.2.2 of [51]. The pressure of 1 kPa is chosen to ensure that the bubbles are oscillating in their linear regime and the obtained resonance frequency is their linear resonance frequency.

## 3 Results

The initial radius of all of the bubbles is chosen to be 1 μm that coincides with sizes that are of interest in medical applications of MBs[52], [53]. To better understand the effect of delays on the bubble radial oscillations, we investigate the changes in their linear resonance frequency varying inter-bubble distances.

**Fig. 3:**
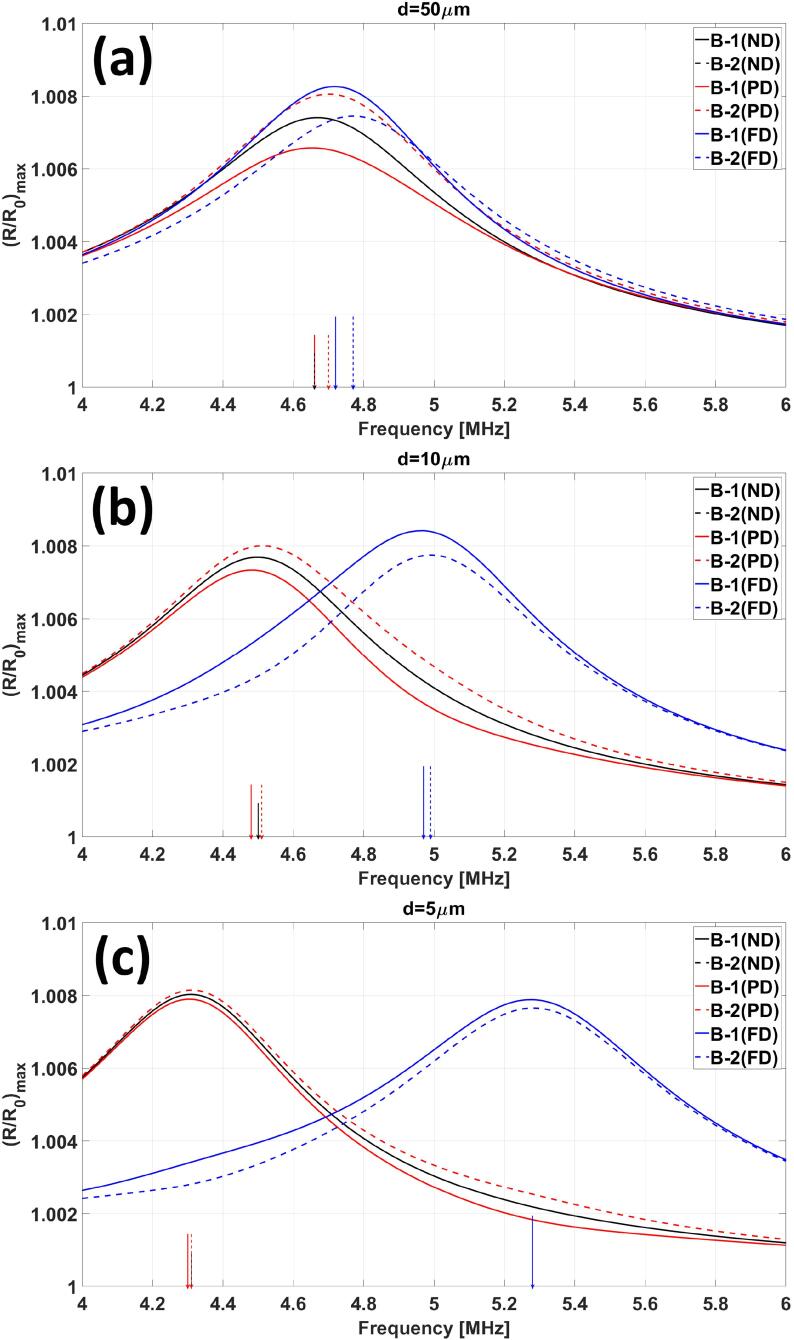
Frequency response curve of 2-MB bubble clusters at distances of 50 μm, 10 μm and 5 μm simulated using the ND (black curves), PD (black curves) and FD (blue curves) models. The colored arrows on the x-axis represent the resonance frequency of the corresponding MB.

**Fig. 4:**
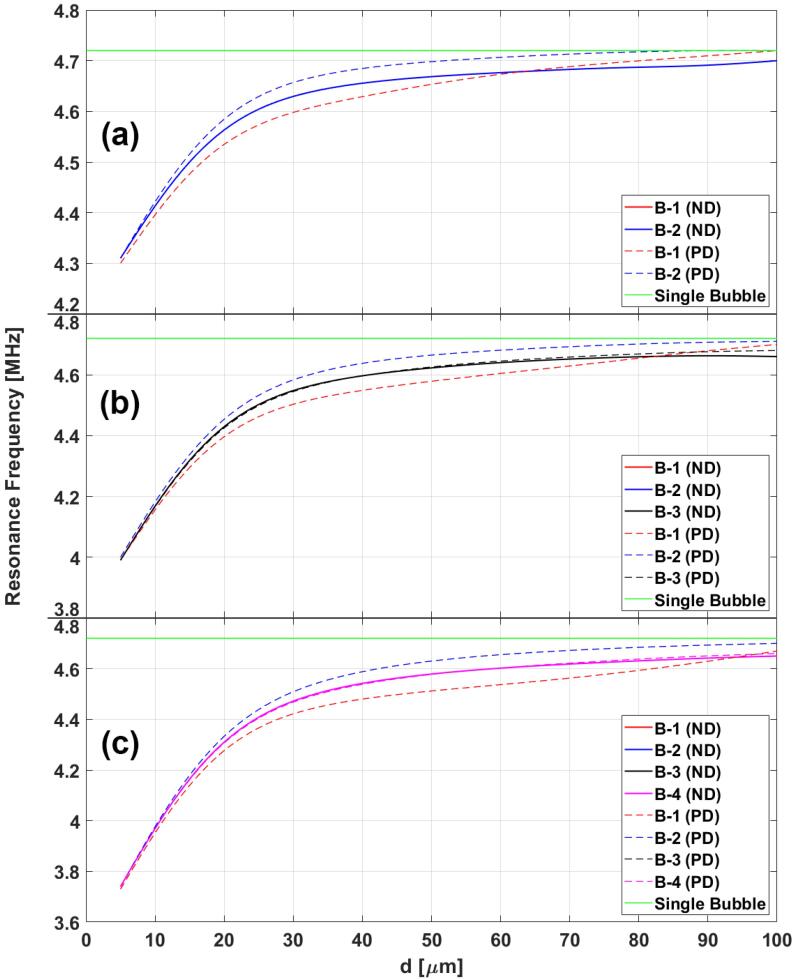
Resonance frequency of 2(a), 3(b) and 4(c) MBs as a function of inter-bubble distances with no delays (ND – solid lines) and only primary delays (PD – dashed line).

**Fig. 5:**
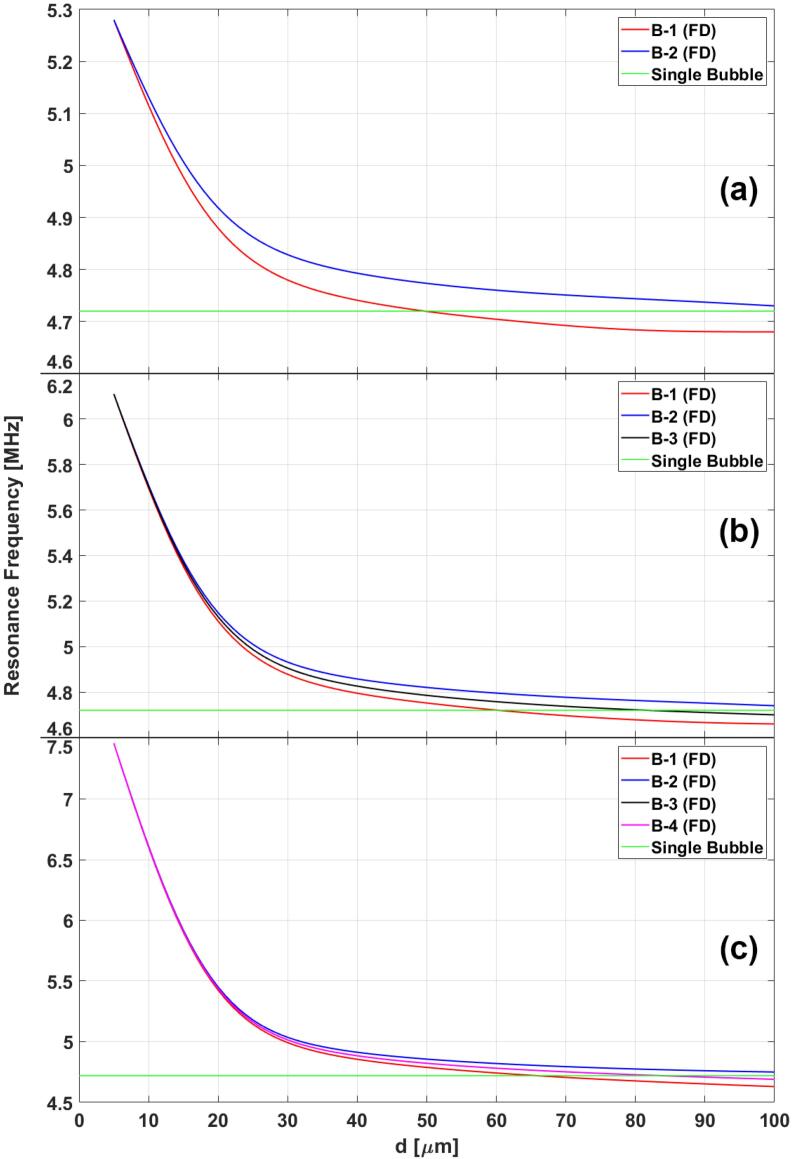
Resonance frequency of 2(a), 3(b) and 4(c) MBs as a function of inter-bubble distances with both primary and secondary delays (FD – full delay) considered.

Fig. 3a shows the frequency response curve of two interacting MBs in for the model with no delay (ND – black), with only primary delays (PD – red) and full delays (FD – blue). Starting from Fig. 3a where MBs are 50 μm apart and moving to Fig. 3b and c MBs are situated closer to each other at distances of 10 μm and 5 μm respectively. Fig. 3 shows that as the MBs get closer to each other, the ND and PD model predict the resonance at lower frequencies where the FD model predicts higher resonance frequency (colored arrows represent the resonance frequency for the respective MBs). In the case of the no delay model, we see that the frequency response curve of the both of the interacting MBs is on top of each other where in the cases of the PD and FD models they are not. Fig. 3 shows that the resonance frequencies of individual MBs within a cluster converge as they are placed closer to each other. This can be seen for both the PD and FD models. This is due to the reduction in primary delays as MBs are placed closer to each other. This effect is seen more clearly in Fig. 4 and Fig. 5 which are discussed next.

Fig. 4 illustrates the MB resonance frequency as a function of inter-bubble distances for the case of 2 (Fig. 4, Fig. 3 (Fig. 4, Fig. 4 (Fig. 4c) MBs with no delay (ND – solid lines) and only the inclusion of primary delays (PD – dashed lines). Fig. 4 shows that all six cases result in a decrease in resonance frequency as the distance between the MBs decreases. The resonance frequency approaches the case of a single isolated MB for larger spacing between the bubbles. Moreover, in the case where delays are ignored (ND – solid lines), all of the MBs have the same resonance frequency (which is to be expected as conditions for all of the MBs is identical). However, Fig. 4 shows that the inclusion of primary delays disrupts this behavior and the resonance frequency of MBs starts to spread out around the case where delays are ignored. MBs closer to the ultrasound source (B-1, Fig. 2) always have the lowest resonance frequency. MBs furthest away from ultrasound source have the highest resonance frequency (B-2 in three cases). Fig. 4 illustrates that the MB resonance frequencies converge to the same value as the MBs as the inter-bubble distances decrease. Therefore, if the MBs are sufficiently close to each other, the effect of primary delays is insignificant and MBs have approximately equal resonance frequencies. Fig. 4 shows that an increase in the number of MBs also results in a decrease in the resonance frequency as well as a sharper decrease in the resonance frequencies with decreasing inter-bubble distances.

**Fig. 6:**
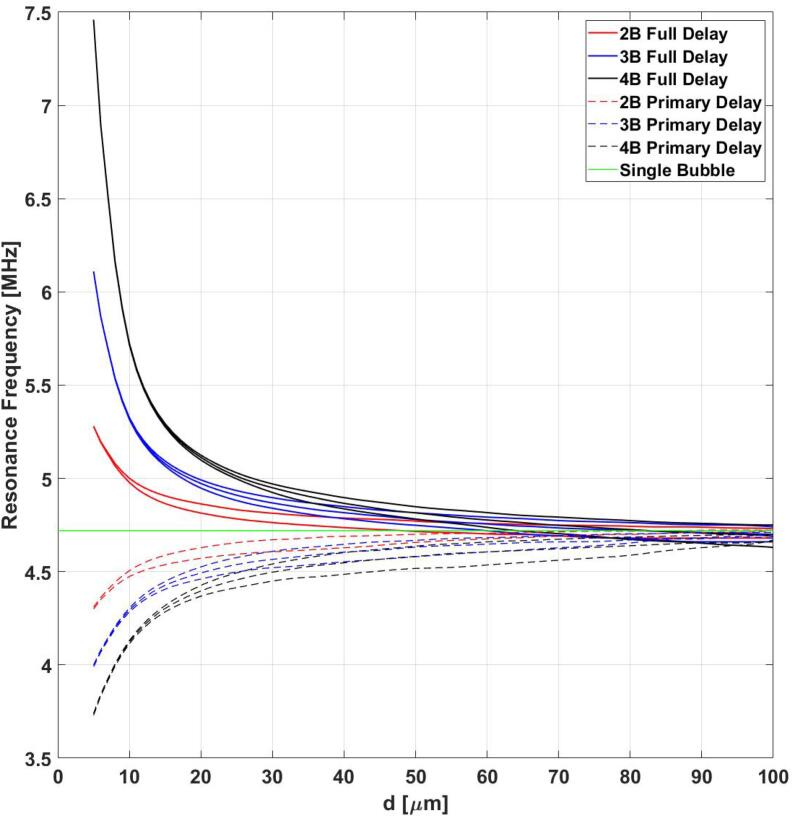
Resonance frequency of 2(black), 3(blue) and 4(black) MBs as a function of inter-bubble distances with only primary delays (PD – dashed lines) and fully delayed (FD – solid line) models.

Fig. 5 shows the calculated resonance frequency in the cases of 2 (Fig. 5, Fig. 3 (Fig. 5, Fig. 4 (Fig. 5c) MBs as a function of inter-bubble distances. The resonance frequency of MBs increases as a function of inter-bubble distances and approaches the resonance frequency of a single isolated MB for large distances. The trend is similar to what is observed in the previous case in Fig. 4. In this case, the primary delays also result in a spread in the resonance frequencies where the closest MB to the ultrasound source (B-1) exhibits the lowest resonance frequency and the furthest MB (B-2) exhibits the highest. Fig. 5 also shows that the effect of primary delays decreases as MBs are situated closer to each other. full delay) included Fig. 6 juxtaposes the case where we only include the primary delays (PD) and the case where both primary and secondary delays (Full Delays – FD) were taken into account. The results in Fig. 6 demonstrate a reversal in the trend observed before: the resonance frequency of MB start to increase with decreasing inter-bubble distances when all the delays are considered. Moreover, the magnitude of resonance change is also more significant when all of the delays are included in the calculations.

## 4 Discussion and conclusions

In this work we investigated the effect of delays in the effective resonance frequency of acoustically coupled MBs.The significance of time delays have been previously shown in the context of the total damping coefficients of acoustically excited MBs [33]. In this work, the authors discovered a reversal trend where inclusion of the secondary delays lead to an increase in damping coefficients with an increasing number of MBs (in a chain). Here we have shown another fundamental property of MBs that reverses its behaviour when secondary delays are considered. Previously, in the absence of secondary delays, it was shown that the resonance frequencies of MBs decrease with increasing concentration [38]. Our results indicate that inclusion of secondary delays reverse this effect and cause the resonance frequency of MBs to increase with increasing concentration. In agreement with previous works [33], [34], this work highlights the importance of inclusion of secondary delays in predicting the dynamics of ultrasonically excited MBs. Moreover, we have shown that primary delays work towards spreading the resonance frequency of the interacting MBs between a minimum and a maximum, where the closest MB to the ultrasound source exhibits the lowest resonance frequency and the furthest MB resonates at the highest resonance frequency. Furthermore, we have shown that if MBs are sufficiently close to each other, the effect of primary delays can be insignificant and ignored. For example, in Fig. 6 we can see that as MBs are placed closer than 20 μm (20 × initial radii of the MBs) to eachother, their resonance frequencies quickly converge to a unique value. However, depending on the tolerances in the application this may be an important consideration that may need to be taken into account. Moreover, we have shown the importance of secondary delays where they result in an increase of resonance frequency with decreasing inter-bubble distances. However, secondary delays have negligible effect on the resonance frequency of MBs when bubbles are sufficiently far away from each other. In Fig. 6 when MBs are 40 μm (40 × initial radii of MBs) or further away from each other, the change in the resonance frequency with respect to single isolated MB case (green line) becomes very small. This is due to the fact that the coupling strength is weak and secondary delays do not have a large enough effect to change the outcome. Secondary delays become more important as MBs approach each other and inter-bubble distances decreases and they work to increase the resonance frequency of MBs. We also have shown that the rate of increase in resonance frequency of MBs is dependent on the number of MBs and with higher number of MBs we observer a higher rate of increase in resonance frequency when MBs approach each other. This is the first time the significance of primary and secondary delay and their effect on resonance frequency of ultrasonically excited coupled MB clusters has been studied in detail. The presented results highlight the importance of the consideration of secondary delays in the study of MB clusters. Moreover, we would expect the results of this study to hold for other MB models, such as for lipid coated MBs and MBs within viscoelastic media. This is because, the interaction term in Eq. (3) (which is the root cause of the observed effects) stays the same. The analysis of this effect in the presence of lipid shelled MBs and viscoelastic media will be the subject of a future study. Generally, we would expect the same trend, however, the magnitude of the resonance shift would differ. In practical applications where resonance frequency of MBs is a determining factor [54] in optimizing the exposure parameters, the failure to estimate $f_{r}$ properly can lead to under optimization and poor performance. Additionally, acoustical attenuation of MBs has been used to estimate the shell properties of the enclosing shell in lipid coated MBs [55], [56]. Here we showed that different concentration of MBs will have different resonance frequencies, therefore, exclusion of secondary delay in shell parameter estimation process can lead to misestimation of the said properties.

## CRediT authorship contribution statement

**Hossein Haghi:** Conceptualization, Methodology, Software, Validation, Data curation, Visualization, Writing – original draft. **Michael C. Kolios:** Supervision, Resources, Funding acquisition, Writing – review & editing.

## Declaration of Competing Interest

The authors declare that they have no known competing financial interests or personal relationships that could have appeared to influence the work reported in this paper.
